# Proteome and Ubiquitome Changes during Rose Petal Senescence

**DOI:** 10.3390/ijms20246108

**Published:** 2019-12-04

**Authors:** Jingyun Lu, Yanjie Xu, Youwei Fan, Yaru Wang, Guifang Zhang, Yue Liang, Chuyan Jiang, Bo Hong, Junping Gao, Chao Ma

**Affiliations:** Beijing Key Laboratory of Development and Quality Control of Ornamental Crops, Department of Ornamental Horticulture, China Agricultural University, Beijing 100193, China; b20143010044@cau.edu.cn (J.L.); xuyanjie@cau.edu.cn (Y.X.); 15652795878@163.com (Y.F.); wangyaru410@163.com (Y.W.); B20183170840@cau.edu.cn (G.Z.); liangyue0124@163.com (Y.L.); cau_jcy@163.com (C.J.); hongbo1203@cau.edu.cn (B.H.); gaojp@cau.edu.cn (J.G.)

**Keywords:** rose hybrid, petal senescence, proteome, ubiquitination

## Abstract

Petal senescence involves numerous programmed changes in biological and biochemical processes. Ubiquitination plays a critical role in protein degradation, a hallmark of organ senescence. Therefore, we investigated changes in the proteome and ubiquitome of senescing rose (*Rosa hybrida*) petals to better understand their involvement in petal senescence. Of 3859 proteins quantified in senescing petals, 1198 were upregulated, and 726 were downregulated during senescence. We identified 2208 ubiquitinated sites, including 384 with increased ubiquitination in 298 proteins and 1035 with decreased ubiquitination in 674 proteins. Gene Ontology (GO) and Kyoto Encyclopedia of Genes and Genomes (KEGG) analyses revealed that proteins related to peptidases in proteolysis and autophagy pathways were enriched in the proteome, suggesting that protein degradation and autophagy play important roles in petal senescence. In addition, many transporter proteins accumulated in senescing petals, and several transport processes were enriched in the ubiquitome, indicating that transport of substances is associated with petal senescence and regulated by ubiquitination. Moreover, several components of the brassinosteroid (BR) biosynthesis and signaling pathways were significantly altered at the protein and ubiquitination levels, implying that BR plays an important role in petal senescence. Our data provide a comprehensive view of rose petal senescence at the posttranslational level.

## 1. Introduction

Petal senescence is the terminal stage of petal development and is generally characterized by wilting, color fading, or petal abscission [1]. Numerous changes in biological and biochemical processes occur during petal senescence, including decreased membrane integrity, degradation of macromolecules and organelles, and remobilization of nutrients, such as carbon, nitrogen, and other mineral elements [2,3,4]. Protein levels in petunia (*Petunia hybrida*) petals decrease dramatically during petal senescence [5], and a massive increase in protease activity has been reported in petunia, daylily (*Hemerocallis hybrid*), and iris (*Iris×hollandica*) [5,6,7]. Phospholipid content also decreases in rose (*Rosa hybrida*), carnation (*Dianthus caryophyllus*), and *Tradescantia* petals during senescence, causing decreased membrane integrity [2,8,9,10]. To recycle nutrients, small molecules, including sucrose, amino acids, and mineral ions, are transported from senescing petals to developing tissues by diverse transporters in the phloem [11]. Moreover, phytohormones play a pivotal role in regulating the progression of senescence, with ethylene and abscisic acid (ABA) accelerating petal senescence, and cytokinin inhibiting this process [2,3]. However, the roles of other hormones in petal senescence are largely unknown.

Petal senescence is regulated at the transcriptional, translational, and posttranslational levels [2,12,13]. Transcriptional regulation of petal senescence has been studied in plants, such as daylily, iris, carnation, and petunia [14,15,16,17], but little is known about the translational and posttranslational regulation of this process. The activity and destiny of proteins are largely dependent on the type of posttranslational modification (PTM) [18]. Posttranslational ubiquitination occurs extensively, playing a critical role in protein degradation and functioning in almost all aspects of plant biology, including hormone signaling, morphogenesis, reproductive processes, and defense responses [19,20,21]. Ubiquitination mediates the breakdown of target proteins via the 26S proteasome system [22] in several enzyme-catalyzed reactions. Ubiquitin is first activated by forming a high-energy thioester bond with a ubiquitin-activating enzyme (E1) and then transferred to a ubiquitin-conjugating enzyme (E2). E2 carries the activated ubiquitin to ubiquitin ligase (E3), which catalyzes the transfer of ubiquitin to target proteins. Finally, the ubiquitinated protein is degraded by the 26S proteasome [19,23]. In addition to protein degradation, ubiquitination can also mediate nonproteolytic events, such as regulation of transcription, chromatin structure, and vesicular trafficking [24]. Despite the important roles of ubiquitination in senescence, high-throughput ubiquitome analysis has only been conducted in petunia in an effort to understand the mechanism of ethylene-induced petal senescence [25].

Rose is an important ornamental plant worldwide, and flower longevity is a key indicator of commodity value. In this study, we used a label-free quantitative strategy involving antibody-based affinity enrichment and high-resolution liquid chromatography-tandem mass spectrometry (LC-MS/MS) analysis to investigate the rose proteome and ubiquitome during petal senescence. We quantified 1924 proteins and 1419 ubiquitination sites that underwent changes during rose petal senescence, providing insight into several pathways related to rose petal senescence, including proteasome and nonproteasome degradation, autophagy, hormone biosynthesis, and signaling.

## 2. Results and Discussion

### 2.1. Expression of Senescence Marker RhSAG12 is Dramatically Increased from Flower Opening Stage 3 to Stage 5

**Figure 1:**
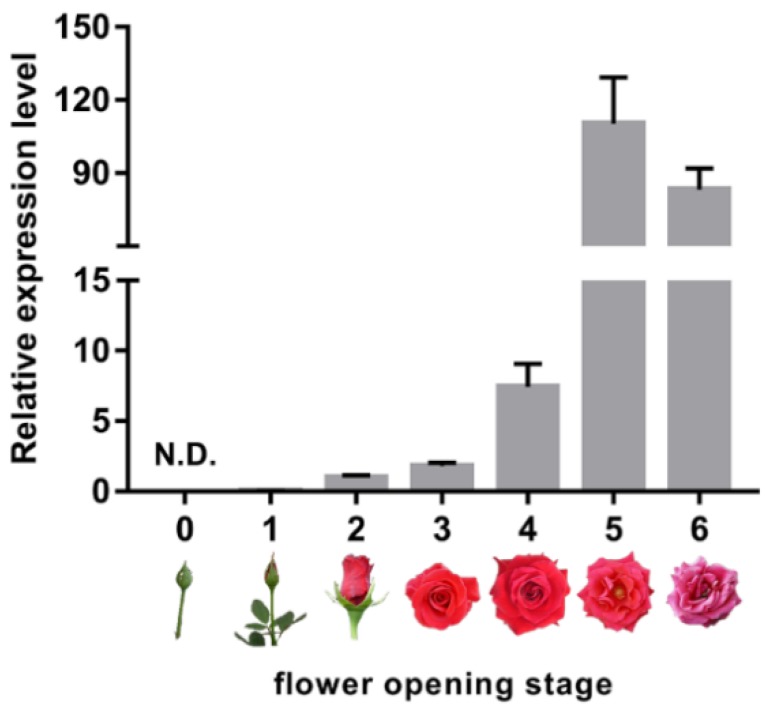
Expression of *RhSAG12* during flower opening. Results are mean values of at least three biological replicates with standard deviations. N.D., no data.

To clarify the senescence-associated status of rose petals, flower opening was divided into seven stages (stages 0–6) as described previously [26] (Figure 1). We then examined the expression of the senescence marker gene *RhSAG12* at stages 0 to 6. The expression of *RhSAG12* was barely detected at stages 0 and 1 (Figure 1). During stages 3 to 5, the transcript abundance of *RhSAG12* dramatically increased by over 50-fold. Flowers in stage 3 displayed no obvious symptoms associated with senescence, while the expression of *RhSAG12* in stage 5 was extremely high. Therefore, flowers at these two stages were chosen to generate proteome and ubiquitome data.

### 2.2. Proteome Profile in Rose Senesced Petal

We investigated changes in the whole proteome at stage 5 compared with stage 3. We identified 5158 proteins, of which 3859 proteins were quantified. A total of 1924 proteins showed dramatic changes in abundance with a threshold of 1.5-fold in stage 5 compared with stage 3. Among them, the expression of 1198 proteins was upregulated, while that of 726 proteins was downregulated in stage 5 compared with stage 3, respectively (Table S1).

**Figure 2:**
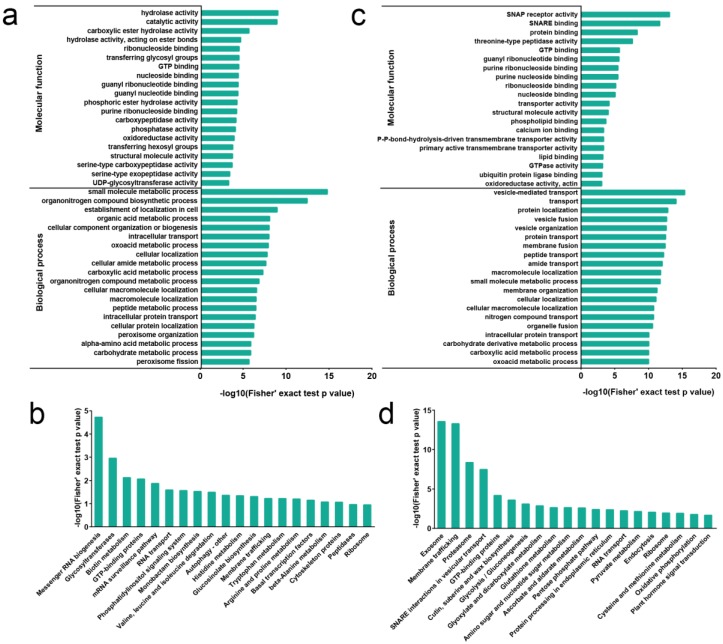
Proteome and ubiquitome functional enrichment analysis. Gene Ontology (GO)-based enrichment analysis of proteins with significantly changed abundance (**a**) and proteins with significantly changed K^ub^ sites (**c**). Kyoto Encyclopedia of Genes and Genomes (KEGG) pathway-based enrichment analysis of proteins with significantly changed abundance (**b**) and proteins with significantly changed K^ub^ sites (**d**).

To evaluate the potential functions of these differentially abundant proteins (DAPs), we performed Gene Ontology (GO) enrichment assays for DAPs (Figure 2a). In the molecular function category, DAPs were enriched in hydrolase activity, catalytic activity, ribonucleoside binding, transferase activity, guanosine triphosphate (GTP) binding, carboxypeptidase activity, phosphatase activity, oxidoreductase activity, serine-type carboxypeptidase activity, and serine-type exopeptidase activity. Upregulated DAPs were enriched in catalytic activity, carboxylic ester hydrolase activity, hydrolase activity, and S-acyltransferase activity, while downregulated DAPs were enriched in hydrolase activity, phosphoric ester hydrolase activity, phosphatase activity, and carboxypeptidase activity (Table S2). These enrichment results suggest that catabolic processes and protein modification have important roles in petal senescence. In addition, proteins related to proteolysis-related peptidase activities were significantly enriched. This is consistent with previous studies demonstrating that organ senescence is accompanied by elevated protein proteolysis in species such as iris and petunia [2,5,7].

In the biological process category, DAPs were enriched in the small molecule metabolic process, organonitrogen compound biosynthetic process, organic acid metabolic process, oxoacid metabolic process, cellular amide metabolic process, carboxylic acid metabolic process, alpha-amino acid metabolic process, and carbohydrate metabolic process (Figure 2a). Upregulated DAPs were enriched in small molecule metabolic process, organonitrogen compound biosynthetic process, and oxoacid metabolic process; downregulated DAPs were enriched in the establishment of localization in the cell, cellular localization, intracellular transport, and cellular amide metabolic process (Table S2). These results suggest that many metabolic processes were influenced during petal senescence.

To further delineate the metabolic pathways participating in petal senescence, we mapped the DAPs against the Kyoto Encyclopedia of Genes and Genomes (KEGG) database (Figure 2b). Eighteen biochemical pathways were significantly enriched (*p* < 0.05) in stage 5 compared to stage 3, including messenger RNA biogenesis, mRNA surveillance pathway, valine, leucine and isoleucine degradation, autophagy, and metabolism of amino acids, such as histidine, tryptophan, arginine, proline, and beta-alanine. Among them, upregulated proteins were significantly enriched in glycosyltransferases, N-glycan biosynthesis, messenger RNA biogenesis, and GTP-binding proteins; downregulated proteins were significantly enriched in messenger RNA biogenesis, glyoxylate and dicarboxylate metabolism, mRNA surveillance pathway, and RNA degradation (Table S2). These data suggest that programmed cell death, amino acid metabolism, and protein degradation play important roles in petal senescence.

### 2.3. Ubiquitome Profile in Rose Senesced Petals

Ubiquitination has critical roles in protein quality control and cell death in eukaryotes [27]. To investigate the involvement of ubiquitination in petal senescence, we quantified protein ubiquitination in petals of stage 5 compared to those of stage 3. We identified 2893 lysine ubiquitination (K^ub^) sites in 1395 proteins, of which 2208 K^ub^ sites in 1142 proteins were quantified. Among these quantified K^ub^ sites and proteins, 384 sites in 298 proteins were identified as upregulated ubiquitination sites, and 1035 sites in 674 proteins were identified as downregulated ubiquitination sites (Table S3).

We conducted GO enrichment analysis to further analyze the function of these proteins with changed K^ub^ sites (Figure 2c, Table S4). In the molecular function category, many transporter-associated activity terms were significantly enriched, including NSF attachment protein (SNAP) receptor activity, soluble NSF attachment protein receptor (SNARE) binding, transporter activity, primary active transmembrane transporter activity, and P-P-bond-hydrolysis-driven transmembrane transporter activity, suggesting that ubiquitination regulates diverse transporter activities. In the biological process category, consistent with the molecular function category, the enriched GO terms were associated with transport processes, including vesicle-mediated transport, protein transport, peptide transport, amide transport, and nitrogen compound transport (Figure 2c).

We used KEGG pathway analysis to compare the proteins with changed K^ub^ sites with metabolic pathways. As shown in Figure 2d, pathways associated with membrane trafficking, proteasome, SNARE interactions in vesicular transport, glutathione metabolism, amino sugar and nucleotide sugar metabolism, ascorbate and aldarate metabolism, pyruvate metabolism, cysteine and methionine metabolism, and plant hormone signal transduction were significantly enriched, suggesting that many primary and secondary metabolites and hormone pathways are regulated by ubiquitination during petal senescence.

**Figure 3:**
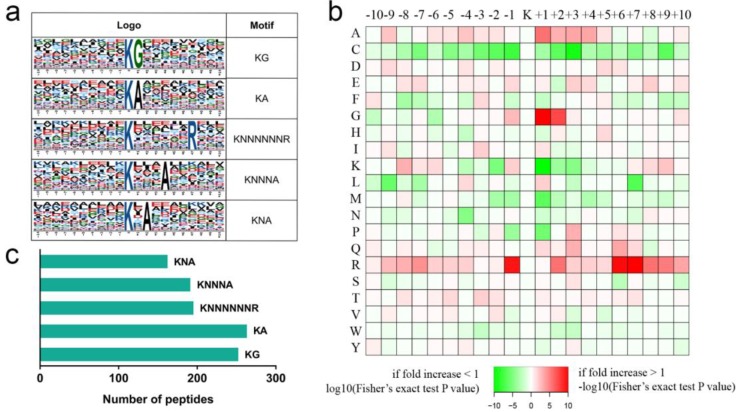
Motif analysis of all identified K^ub^ sites in rose. (**a**) Five ubiquitination motifs and the conservation of K^ub^ sites. (**b**) Amino acid sequence properties of ubiquitination sites. The heat map shows significant position-specific underrepresentation or overrepresentation of amino acids flanking the modification sites. (**c**) Number of identified peptides containing ubiquitinated lysine in each motif.

We used the Motif-X program to determine position-specific frequencies of amino acid residues surrounding all K^ub^ sites identified in rose petals. Among the 2893 K^ub^ sites, 1058 were assigned to five conserved motifs, accounting for 37% of the K^ub^ sites identified (Figure 3, Table S5): 251 K^ub^G, 262 K^ub^A, 194 K^ub^NNNNNNR, 190 K^ub^NNNA, and 161 K^ub^NA motifs (N indicates any amino acid; Figure 3c). Previous studies have reported K^ub^NNNA and K^ub^NA sites in rice (*Oryza sativa*) and wheat (*Triticum aestivum*) [28,29], while the other three sites were novel in rose. In addition, we observed that alanine (A) was enriched at the +1, +2, and +4 positions around K^ub^. Previous studies in rice and wheat also demonstrated that most conserved ubiquitinated motifs possess alanine (A) [28,29,30].

### 2.4. Association between the Global Proteome and Ubiquitome

**Figure 4:**
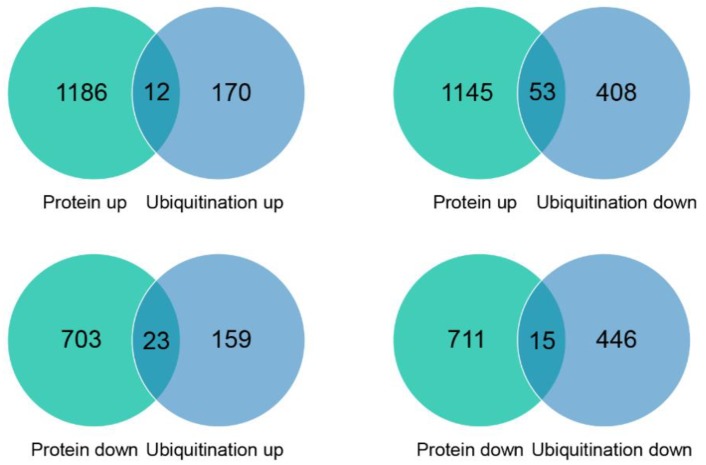
Venn diagrams showing common proteins significantly up- or downregulated at the protein and ubiquitination levels.

A well-established function of ubiquitination is mediating protein degradation via the 26S proteasome. To investigate interactions between the proteome and ubiquitome, we compared DAPs with ubiquitinated proteins. We screened our ubiquitome, defining proteins in which all K^ub^ sites showed increased levels of ubiquitination in stage 5 petals compared with stage 3 petals as upregulated ubiquitinated proteins, and proteins in which all K^ub^ sites showed decreased levels of ubiquitination in stage 5 petals compared with stage 3 petals as downregulated ubiquitinated proteins. The abundance of 23 upregulated ubiquitinated proteins was decreased, including putative vesicle transport v-SNARE 12 (XP_024194691) and cytokinin signaling component AHP1 (XP_024191812) (Figure 4, Table S6). The abundance of 53 downregulated ubiquitinated proteins was increased (Figure 4, Table S6), including putative sucrose transport protein SUC3 (XP_024192899), receptor of strigolactone D14 (XP_024183944), and salicylic acid-responsive transcription factor TGA2.3-like (XP_024181993) (Figure 4, Table S6). Many ubiquitinated proteins could not be detected in our study as they had presumably already been degraded by the 26S proteasome. Therefore, the ubiquitome does not reflect the status of protein degradation. Further spatial and temporal ubiquitome analyses of petal senescence will explain the negative association between the proteome and ubiquitome. In addition to its role in protein degradation, mono- or poly-ubiquitination as types of protein modification alter biochemical properties and subcellular protein localization [31]. The technology used in this study cannot distinguish the number of ubiquitins added to K residues. Therefore, our study could not dissect the functions of protein modification by ubiquitination in senescence.

### 2.5. Transcription Factors and Protein Kinases

Plants have evolved complex transcriptional networks that regulate senescence; transcription factors act as master switches of transcriptional reprogramming in this process [32]. We identified 23 transcription factors in the rose petal senescence proteome, including four putative bHLHs (including MYC2 and PRE1), four bZIPs, two nuclear factors, two WRKYs, one NAC, and one HD-Zip transcription factor (Table S7). Among these, 17 showed increased abundance, and 6 showed reduced abundance in stage 5 petals compared to stage 3 petals. The petunia bHLH transcription factors PhFBH4 and AN1 promote petal senescence by activating ethylene biosynthesis genes, and silencing of these two genes results in delayed petal senescence [33,34]. In *Arabidopsis* (*Arabidopsis thaliana*), a master bZIP transcription factor in ABA signaling, ABI5, activates the expression of the major senescence-promoting NAC transcription factor ORE1 during dark-induced leaf senescence [35]. *Arabidopsis* AtWRKY53 positively regulates leaf senescence by activating the expression of SAG12 and ORE9 [36], while another WRKY protein, AtWRKY57, negatively regulates senescence by repressing the expression of *SAG* genes [37]. Members of the plant-specific NAM/ATAF/CUC (NAC) transcription factor family are involved in petal senescence in several plant species. In morning glory (*Ipomoea nil*), the silencing of EPHEMERAL1, a NAC transcription factor, suppresses petal senescence [38]. In rose, two HD-Zip proteins, RhHB1 and RhHB6, regulate petal senescence positively and negatively, respectively [39,40]. In petunia, PhHD-Zip regulates petal senescence by promoting ABA biosynthesis [41].

We detected 10 greatly changed K^ub^ sites in eight transcription factors in senescing rose petals (Table S7). Notably, ubiquitination levels of two transcription factors, bZIP family member TGA2.3-like (XP_024181993) and NAC family member NTM-like9 (XP_024169726), were downregulated at stage 5 compared to stage 3, while their proteins accumulated at stage 5, suggesting that these two transcription factors are tightly regulated by ubiquitination during petal senescence. Previous studies have demonstrated that ubiquitination is involved in regulating homeostasis of transcription factors during senescence. In *Arabidopsis*, a RING-type ubiquitin E3 ligase NLA participated in leaf senescence by ubiquitinating and degrading AtNAC092 under nitrogen-starvation conditions [42]. In apple, a BTB protein, MdBT2, interacted with a senescence-promoting transcription factor, MdbHLH93, and induced degradation and ubiquitination of MdbHLH93 in ABA-modulated leaf senescence [43].

Protein kinases (PKs) are essential signaling regulators of senescence [44]. We identified 40 PKs in the rose proteome of which the abundance of 24 was increased while that of 16 was decreased in stage 5 petals compared to stage 3 petals (Table S7). Among these PKs, receptor-like kinases (RLKs) were the largest group (16 of 40; Table S7). In soybean (*Glycine max*), a leucine-rich repeat receptor-like protein kinase promotes leaf senescence by repressing cytokinin signaling and inducing auxin and ethylene signaling [45]. In *Arabidopsis*, mutation of *AtRPK1*, encoding a membrane-bound receptor protein kinase, delays age- and ABA-induced senescence [46]. In rice, overexpression of *OsSIK2*, encoding a receptor-like kinase, results in delayed dark-induced leaf senescence, while the *sik2* mutant shows the opposite phenotype [47]. In addition, five putative mitogen-activated protein kinases (MPKs) were identified in the rose proteome; all of them accumulated considerably in stage 5 compared to stage 3 (Table S7). In *Arabidopsis*, the MKK9-MPK6 cascade plays an important role in regulating leaf senescence, with *mkk9* and *mpk6* mutants showing delayed leaf senescence [48]. In maize (*Zea mays*), functional analysis of a member of the MPK kinase (MAPKK) family, ZmMEK1, indicated that ZmMEK1 promotes leaf senescence [49].

In the rose ubiquitome, 48 K^ub^ sites in 31 PKs were significantly changed (Table S7) during petal senescence. Among these 31 PKs, ubiquitination levels of 6 PKs, including three RLKs, were upregulated, while those of 19 PKs, including 12 RLKs and four threonine kinase-like proteins were downregulated. In addition, we found three PKs with decreased ubiquitination levels that accumulated in stage 5 compared to stage 3: one RLK (XP_024165045), one cyclin-dependent kinase (XP_024191618), and one casein kinase (XP_024193263). To date, few studies have explored the ubiquitination of PKs involved in senescence. Therefore, functional characterization of these PKs and identification of their interactive E3 ligases may shed light on their roles in petal senescence.

### 2.6. Proteasome and Non-Proteasome Pathways

Ubiquitin-proteasome and protease systems are responsible for protein degradation during senescence [50]. Three putative ubiquitin-conjugating enzymes (E2s), 17 ubiquitin ligases (E3s), and two 26S proteasome subunits were upregulated in the rose petal proteome at stage 5 compared to stage 3 (Table S8). In petunia, silencing of *PhXB3* encoding a RING zinc-finger E3 ligase significantly prolongs flower life [51]. In addition, multiple components of the proteasome pathway associated with flower senescence have been identified via transcriptome approaches in carnation, morning glory, and petunia [16,52,53], although their exact roles in petal senescence remain unknown.

We also observed that 15 putative proteases were upregulated in stage 5 compared to stage 3, including four serine proteases, six aspartyl proteases, one caspase, and four metalloproteases (Table S8). In daylily and iris petals, the activity of protease system-related proteins increases before visible petal senescence [6,7]. In iris flowers, inhibiting serine protease activity by treatment with the serine protease inhibitor AEBSF delays flower senescence [7]. In sandersonia (*Sandersonia aurantiaca*), decreasing endoprotease activity via the cysteine protease inhibitor 2,2′-dipyridyl delays petal wilting [54]. In carnation, the expression of the caspase gene *DCCP1* increases with the onset of ethylene production and senescence in petals, indicating that DCCP1 might be involved in ethylene-induced senescence [55]. Moreover, through omics approaches, many proteases involved in petal senescence have been identified in ornamental crops, such as alstroemeria (*Alstroemeria pelegrina*), daylily, iris, and petunia [14,15,25,56].

Intriguingly, the abundance of three chloroplast-localized serine proteases (XP_024184581, XP_024160425, and XP_024173197) and three chloroplast-localized metalloproteases (XP_024180294, XP_024187022, and XP_024188345) was upregulated in stage 5 compared to stage 3. In alstroemeria and iris, such chloroplast-localized proteases were also found to be associated with petal senescence [15,56]. It is well known that no chloroplasts exist in petal cells. Identification of these proteases implies that they might function in the degradation of other plastids, such as leukoplastids and chromoplastids.

### 2.7. Autophagy Pathway

**Figure 5:**
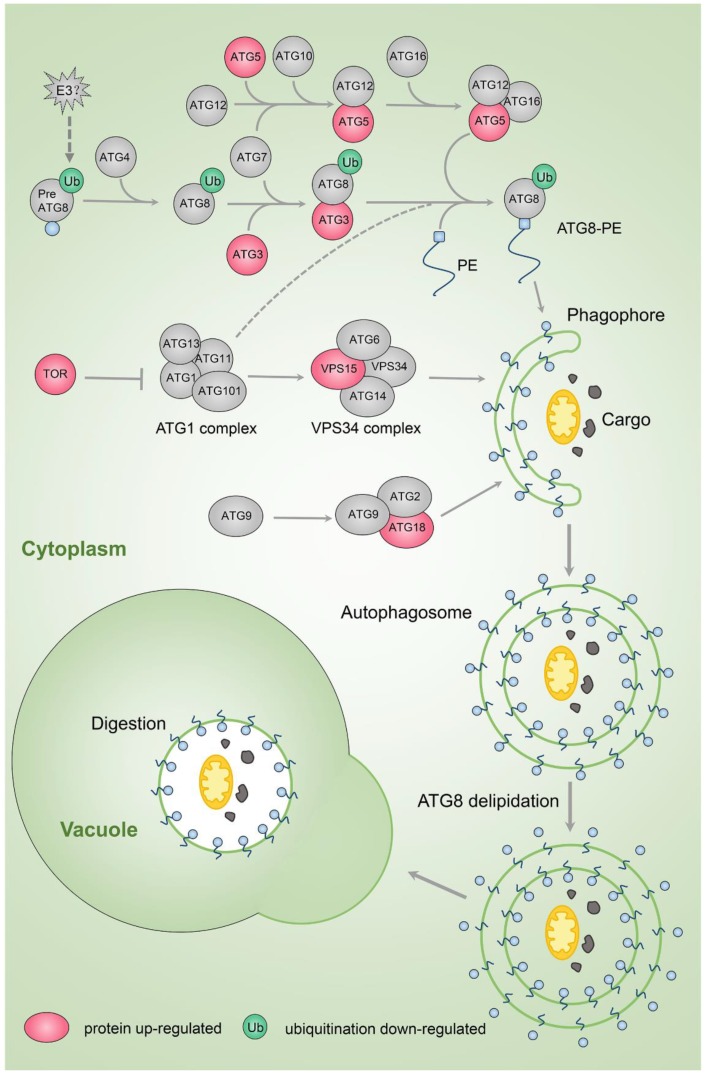
Changes in protein and ubiquitination levels of autophagy pathway components during rose petal senescence. PE, phosphoethanolamine.

Autophagy is one of the main mechanisms for removing and recycling useless or damaged cell components via vacuoles [57]. The relationship between autophagy and senescence is sophisticated. High levels of autophagy activity lead to cell death, while a certain degree of autophagy is required for discarding useless cell components to maintain cell longevity [57]. In the rose petal proteome, we found that several putative autophagy (ATG) proteins, including ATG3 (XP_024194784), ATG5 (XP_024173541), ATG9 (XP_024162099), and ATG18a (XP_024164993), and autophagy-related protein kinases VPS15 (XP_024184320) and TOR1 (XP_024187643) were upregulated at the protein level in stage 5 compared to stage 3 (Table S8, Figure 5). These results indicate that the autophagy pathway is activated during petal senescence in rose, although TOR1 is an inhibitor of autophagy onset. In the autophagy pathway, ATG8 binds autophagy adaptors that facilitate autophagosome assembly and maturation; however, ATG8 also associates with autophagy receptors that sequester cargo into the forming phagophore [58]. Ubiquitination levels of ATG8f (XP_024176024, Lys-70) were reduced in stage 5 relative to stage 3 petals (Table S8), suggesting that ubiquitination might regulate autophagy during petal senescence.

An autophagy-like phenomenon has been observed in senescing leaves, with the transcription of many autophagy-related genes induced [59,60]. Molecular clues suggest that autophagy is also involved in petal senescence. Expression of *ATG* genes increases during petal senescence in morning glory and petunia [61,62,63]. In petunia, a monodansylcadaverine (MDC) signal indicating autophagic structures appears during petal senescence [63]; while in morning glory, application of 3-methyladenine (3-MA), an inhibitor of autophagic structure formation, accelerates senescence [61].

### 2.8. Hormone Biosynthesis and Signaling Pathways

**Figure 6:**
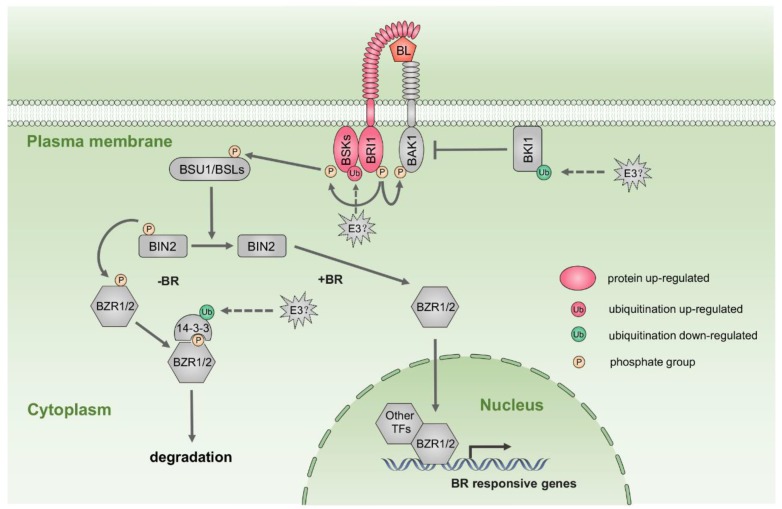
Changes in protein and ubiquitination levels of brassinosteroid (BR) signaling pathway components during rose petal senescence. TF, transcription factor; BL, brassinolide.

Phytohormones act as internal cues to initiate petal senescence [3]. Our results showed that many components of hormone biosynthesis and signaling were significantly changed at the protein or ubiquitination levels in stage 5 compared to stage 3 (Table S9). We identified several enzymes involved in brassinosteroid (BR) biosynthesis and metabolism from proteome data. The abundance of BAS1, a BR-inactivating protein, was increased in stage 5 compared to stage 3 petals. In addition, we detected K^ub^ sites in two enzymes participating in BR biosynthesis, DWF1 (Lys-510, Lys-511) and CYP716A1 (Lys-136). Among proteins related to the BR signaling pathway, the putative receptor BRI1 (XP_024170330) was more abundant in stage 5 than in stage 3 petals (Figure 6, Table S9). The accumulation of putative BR SIGNALING KINASE (BSK) proteins, including BSK1 (XP_024165639), BSK5 (XP_024159144), and BSK6 (XP_024190715), was also substantially increased at stage 5 (Figure 6, Table S9). PRE1 (XP_024179999), a transcription factor in the BR signaling pathway, also accumulated in stage 5 (Table S9).

Ubiquitination at a K^ub^ site in putative BSK1 (Lys-78) was increased at stage 5 compared to stage 3, and ubiquitination levels of K^ub^ sites in putative BKI1 (XP_024166024, Lys-222; Figure 6, Table S9) and putative 14-3-3 (XP_024169629, Lys-123; Figure 6, Table S7) were reduced in stage 5 relative to stage 3 petals. In *Arabidopsis*, BKI1 blocks BR signaling in the absence of BR by interacting with BRI1 [64]; 14-3-3 protein binds to the phosphorylated BR positive regulator BZR1, resulting in nuclear export and degradation of BZR1 by the proteasome pathway [64]. These results suggest that BR signaling might play an important role in petal senescence. In *Arabidopsis*, the BR receptor mutant *bri1* shows a premature leaf senescence phenotype [65]. In mung bean (*Phaseolus radiatus*) and papaya (*Carica papaya*), exogenous BR accelerates leaf senescence [66,67,68]. However, the effects of BRs on petal senescence are largely unknown.

Ethylene plays a critical role in promoting senescence [3,69]. *S*-adenosyl-met (SAM) is a precursor in the ethylene biosynthesis pathway whose formation is catalyzed by *S*-adenosyl-Met synthetase (SAMS). We identified five K^ub^ sites in two putative SAMSs (XP_024193334, Lys-153, Lys-333, Lys-388, Lys-391; XP_024191015, Lys-390). In addition, SAMS (XP_024188677) was upregulated at the protein level. Petunia SAMSs show increased ubiquitination and decreased protein levels during petal senescence, suggesting possible negative feedback regulation of SAMSs by ethylene [25]. In the ethylene signaling pathway, the transduction of extranuclear signals to downstream gene expression is mainly mediated by the central transcription factor EIN3. We detected the downregulation of one K^ub^ sites in EIN3 (XP_024185087, Lys-123) (Table S9). In *Arabidopsis*, ubiquitination and degradation of EIN3 are mediated by two F-box proteins, EBF1 and EBF2 [70]. Taken together, our results suggest that ethylene biosynthesis and signaling pathways are activated during petal senescence, consistent with the results of a previous study [39].

In the jasmonate (JA) biosynthesis pathway, JAR1 catalyzes the conversion of JA to its active form JA-Ile [71]. In this study, a putative JAR1 (XP_024176101) accumulated in stage 5 petals (Table S9) and the ubiquitination levels of all K^ub^ sites (Lys-29, Lys-90, Lys-154, and Lys-506) in JAR1 were reduced at this stage, suggesting that JA-Ile abundance is positively regulated during petal senescence via the ubiquitination pathway.

In the JA signaling pathway, MYC2 activates the JA response by promoting the expression of JA-responsive genes [72]. The effects of JA on petal senescence are largely unknown. Here, we found that a putative MYC2 accumulated in stage 5 petals but was not detected in stage 3 petals (Table S9). In *Arabidopsis*, the application of methyl jasmonate (MeJA) accelerates leaf senescence, and genes involved in the JA biosynthesis pathway are upregulated during leaf senescence [73]. In addition, mutation of JA signaling components greatly affects leaf senescence [37,74]. In iris flowers, JA treatment slightly delays tepal senescence [75], but in orchid (*Phalaenopsis hybrid*) and Lily (*Lilium* L.), endogenous JA content does not change during flower senescence [3].

### 2.9. Transporter Activity

During petal senescence, macromolecules are degraded and remobilized in a process accompanied by enhanced transporter activities [2]. The abundance of many transporters, including potassium, copper, magnesium, sugar, GDP-mannose, oligopeptide, lysine histidine, and ABC transporters, was increased at stage 5 compared to stage 3 in the senescing rose petal proteome (Table 1). Ubiquitination levels of 44 K^ub^ sites in 31 transporter proteins were significantly changed at stage 5 compared to stage 3 (Table 2). Intriguingly, ubiquitination levels of most of these transporter proteins (28 K^ub^ sites in 22 proteins) were downregulated, and four of these showed greater accumulation at stage 5, including putative transporters of potassium, sucrose, lysine histidine, and molybdate-anion. Therefore, ubiquitination and de novo synthesis might jointly regulate the increase in transporter proteins during petal senescence. In morning glory, 60% of the K^+^ content, about 50% of the Mg^2+^ content, and about 25% of the Ca^2+^ content is transferred to other parts of the plant during petal senescence [76]. In petunia, potassium and phosphorus contents decrease [77,78], and transcripts of a phosphate transporter, *PhPT*, accumulate in senescing corollas [78]. Expression of Fe transporter genes is induced in senescing rose petals [79]. Moreover, many transporters related to petal senescence were identified via transcriptome approaches in plant species, such as alstroemeria, carnation, and iris [15,16,56]. These results suggest that enhanced transporter activity might play critical roles in petal senescence.

A putative auxin influx protein, LAX3 (XP_024166942), accumulated in stage 5 compared to stage 3. In addition, ubiquitination levels of three putative auxin efflux proteins, PIN3 (XP_024193331, Lys-204), ABCB1 (XP_024186868, Lys-66, Lys-76, Lys-731, Lys-1208), and ABCB19 (XP_024181861, Lys-1066), were reduced during senescence (Table 2). In *Arabidopsis*, PTMs, including phosphorylation and ubiquitination, are involved in regulating apical-basal polarity and turnover of PINs [80]. In petunia, ubiquitination of ABCB proteins was changed in the ubiquitome data during ethylene-induced corolla senescence [25]. Taken together, our results suggest that auxin transport, including both influx and efflux, participate in petal senescence.

## 3. Conclusions

We performed a global investigation of the proteome and ubiquitome of senescing rose petals. Proteins related to peptidase in proteolysis and autophagy pathways were enriched in the proteome during petal senescence, suggesting that protein degradation and autophagy might play important roles in petal senescence. Many transporter proteins accumulated in senescing petals and several transport processes were enriched in the ubiquitome of senescing petals, indicating that the transport of substances is associated with petal senescence and is regulated by ubiquitination. In addition, several components of the BR biosynthesis and signaling pathways were significantly altered at the protein and ubiquitination levels, implying that BR plays an important role in rose petal senescence.

## 4. Materials and Methods

### 4.1. Plant Material

Rose (*Rosa hybrida* ‘Samantha’) flowers were harvested at flower opening stages 0–6 [26] from a local greenhouse in Beijing, placed immediately in deionized water, and delivered to the laboratory within 2 h. For gene expression assays, petals from the middle whorl of flowers at each stage were collected and frozen at −80 °C. For proteome and ubiquitome assays, petals from the middle whorl of seven flowers at stage 3 and seven flowers at stage 5 were collected and frozen at −80 °C.

### 4.2. Total RNA Extraction and Quantitative RT-PCR

Total RNA was extracted using the hot borate method as previously described [81], followed by RNase-free DNase I (Promega) treatment to remove contaminating genomic DNA. For quantitative RT-PCR, 1 μg of total RNA was used to synthesize cDNA using M-MLV reverse transcriptase (Promega) and 2 μL of cDNA was then used as the template in each 20-μL quantitative RT-PCR reaction using a StepOnePlus^TM^ real-time PCR system (Applied Biosystems) with KAPATM SYBR^®^ FAST quantitative PCR kits (Kapa Biosystems). The expression of *RhSAG12* (GenBank accession number XM_024301607) was analyzed. The rose *Ubiquitin2* gene *RhUBI2* was used as an internal control [82]. Three biological replicates were performed.

### 4.3. Protein Extraction

Petal samples were ground in liquid nitrogen and transferred to 5-mL centrifuge tubes. Fourfold volume of lysis buffer (8 M urea, 1% Triton X-100, 10 mM dithiothreitol, 1% protease inhibitor (Calbiochem, Germany), deubiquitinase inhibitors PR-619 (Sigma, USA), and 2 mM EDTA was added to the cell powder, and mixtures were sonicated three times on ice using a high-intensity ultrasonic processor (Scientz). Sonicated products were centrifuged at 20,000× *g* at 4°C for 10 min to remove remaining debris. The supernatant was collected, and a final concentration of 20% (*v*/*v*) trichloroacetic acid (TCA) was added. The mixtures were kept at 4 °C for 2 h and then centrifuged at 12,000× *g* at 4 °C for 3 min; the supernatant was discarded, and the precipitate was washed three times with precooled acetone. Finally, the precipitate was dissolved in urea (8 M), and the protein concentration was determined with a BCA kit (Beyotime Biotechnology, Shanghai, China) according to the manufacturer’s instructions.

### 4.4. Trypsin Digestion

Dithiothreitol (final concentration of 5 mM) was added to the protein solution and reduced for 30 min at 56 °C. Iodoacetamide (final concentration of 11 mM) was then added and kept in darkness for 15 min at 23 °C. Protein samples were diluted with NH_4_HCO_3_ (100 mM) to a urea concentration of <2 M. Protein samples were first digested using a 1:50 trypsin/protein ratio at 37 °C overnight and then digested using a 1:100 trypsin/protein ratio for 4 h.

### 4.5. HPLC Fractionation

Tryptic peptides were fractionated by high pH reverse-phase HPLC using an Agilent 300 extend C18 column (5-μm particles, 4.6-mm i.d., and 250-mm length) for proteome analysis and a Thermo betasil C18 column (5-μm particles, 10-mm i.d., and 250-mm length) for ubiquitome analysis. Briefly, peptides were first separated with a gradient of 8% to 32% acetonitrile (pH 9.0) over 60 min into 60 fractions. The peptides were then combined into 10 fractions (proteome assay) or 4 fractions (ubiquitome assay) and dried by vacuum freezing.

### 4.6. Affinity Enrichment

Since ubiquitinated proteins have a distinct di-Gly remnant (K-ε-GG), a high-quality anti-K-ε-GG antibody (PTM Biolabs) was used to enrich ubiquitinated proteins. Tryptic peptides were dissolved in immunoprecipitation buffer (100 mM NaCl, 1 mM EDTA, 50 mM Tris-HCl, and 0.5% NP-40, pH 8.0). Dissolved peptides were incubated with prewashed anti-K-ε-GG antibody beads (lot number PTM1104, PTM Bio) and gently shaken at 4 °C overnight. The beads were then washed four times with immunoprecipitation buffer and twice with deionized water. Finally, the bound peptides were eluted three times, with 0.1% trifluoroacetic acid, and the eluent was dried by vacuum freezing. For LC-MS/MS analysis, the resulting peptides were desalted with C18 ZipTips (Millipore) according to the manufacturer’s instructions.

### 4.7. LC-MS/MS Analysis

Tryptic peptides were dissolved in 0.1% formic acid (solvent A) and loaded onto a reversed-phase analytical column (15-cm length and 75-μm i.d.). A gradient was achieved with solvent B (0.1% formic acid in 90% acetonitrile) increasing from 5% to 25% in 26 min, 25% to 40% in 8 min, and 25% to 80% in 3 min, and then held at 80% for the last 3 min, all at a constant flow rate of 350 nL/min on an EASY-nLC 1000 ultraperformance liquid chromatography (UPLC) system (ThermoFisher Scientific).

Peptides were subjected to a nanospray ionization source followed by tandem mass spectrometry (MS/MS) in a Q Exactive^TM^ Plus (Thermo) coupled online to the UPLC. The electrospray voltage applied was 2.0 kV. The m/z scan range was 350 to 1800 for a full scan. Intact peptides were detected in the Orbitrap at a resolution of 70,000. Peptides were selected for MS/MS using a normalized collision energy (NCE) setting of 28, and fragments were detected in the Orbitrap at a resolution of 17,500. A data-dependent procedure that alternated between one MS scan and 20 MS/MS scans was applied with 15.0-s dynamic exclusion. Automatic gain control was set at 5E4. The mass spectrometry proteomics data have been deposited to the ProteomeXchange Consortium via the PRIDE partner repository with the dataset identifier PXD016490.

### 4.8. Database Search

MS/MS data were processed using the Maxquant search engine (v.1.5.2.8, Germany). Tandem mass spectra were searched against the rose (*Rosa chinensis*) genome database (https://lipm-browsers.toulouse.inra.fr/pub/RchiOBHm-V2/) concatenated with a reverse decoy database.

For proteomic peptides, trypsin/P was specified as the cleavage enzyme allowing up to two missing cleavages. The minimum length of peptides was set as 7, and the number of maximum modifications was set as 5. The mass tolerance for precursor ions was set as 20 ppm in the first search and 5 ppm in the main search, and the mass tolerance for fragment ions was set as 0.02 Da. Carbamidomethyl on Cys was specified as the fixed modification. Oxidation on Met and acetylation on the N terminus of proteins were specified as variable modifications. The label-free quantification method was LFQ, false discovery rate (FDR) was adjusted to < 1%.

For ubiquitinated peptides, trypsin/P was specified as the cleavage enzyme allowing up to four missing cleavages. The minimum length of peptides was set as 7, and the number of maximum labeled amino acids was set as 5. The mass tolerance for precursor ions was set as 20 ppm in the first search and 5 ppm in the main search, and the mass tolerance for fragment ions was set as 0.02 Da. Carbamidomethyl on Cys was specified as the fixed modification. Gly on Lys modification, oxidation on Met, and acetylation on the N terminus of proteins were specified as variable modifications. The label-free quantification method was LFQ, FDR was adjusted to < 1%, and the minimum score for modified peptides was set to > 40.

### 4.9. Bioinformatics Analysis

Bioinformatic analysis was performed according to previously described methods [25,28]. Gene Ontology (GO) annotation was derived from the UniProt-GOA database (www.http://www.ebi.ac.uk/GOA/). Proteins were classified by GO annotation into three categories: biological process, cellular compartment, and molecular function. The KEGG database was used to annotate protein pathways.

Ten amino acids upstream and downstream of the K^ub^ sites in all protein sequences were chosen for analyzing sequence properties of ubiquitinated sites using Motif-X software (http://motif-x.med.harvard.edu/motif-x.html). All database protein sequences were used as a background database, and other parameters were set to default values. Setting parameters for motif searching using Motif-X were 20 occurrences and a Bonferroni-corrected *p*-value of 0.000001.

## Figures and Tables

**Table 1:** Transporters with upregulated abundance in the rose petal senescence proteome.

Protein Accession	Protein Description	Mol. Weight (kDa)
XP_024163091.1	Copper transporter 5.1	15.9
XP_024180604.1	Potassium transporter 4	87.8
XP_024185106.1	Magnesium transporter MRS2-1	50.6
XP_024169378.1	Molybdate-anion transporter-like	50.6
XP_024165719.1	Sugar transporter ERD6-like 7	36.8
XP_024166942.1	Auxin transporter-like protein 3 LAX3	52.9
XP_024170862.1	Oligopeptide transporter 7	84.5
XP_024173227.1	GDP-mannose transporter GONST3	59.0
XP_024183764.1	Oligopeptide transporter 4-like	83.0
XP_024165085.1	Lysine histidine transporter-like 8	58.6
XP_024182501.1	Lysine histidine transporter-like 8	60.4
XP_024179150.1	ABC transporter I family member 1	25.7
XP_024160504.1	ABC transporter B family member 25	79.3
XP_024158019.1	ABC transporter A family member 2-like	106.4
XP_024167415.1	ABC transporter C family member 5	170.6
XP_024189449.1	Putative ABC transporter C family member 15 isoform X1	169.6
XP_024190413.1	ABC transporter G family member 15-like	75.4
XP_024191990.1	ABC transporter I family member 17 isoform X2	29.5

**Table 2:** Transporters with significant changes in K ubiquitination in senescing rose petals.

Protein Accession	Position	Protein Description	Modified Sequence	Regulation Type
XP_024166523.1	1271	ABC transporter C family member 14-like	APPTNWPTHGNVELK(1)DLQVR	Up
XP_024167390.1	215	Sugar transporter ERD6-like 7	HK(1)EFEVALQK	Up
XP_024196922.1	254	Equilibrative nucleotide transporter 1	ELK(1)GPLTGSVLR	Up
XP_024158774.1	1314	ABC transporter C family member 2-like	ILIDGCDIGK(1)FGLEDLRK	Up
XP_024176720.1	876	ABC transporter C family member 10-like	LK(1)GNKGDQLIK	Up
XP_024176720.1	879	ABC transporter C family member 10-like	GNK(1)GDQLIK	Up
XP_024163367.1	603	Sulfate transporter 3.1-like isoform X3	FMLHTTK(0.997)SDPVK(0.003)EEPGTWNNV	Up
XP_024186868.1	547	ABC transporter B family member 1	VANAHSFIVK(1)LPDGFDTQVGER	Up
XP_024173043.1	1356	ABC transporter C family member 10-like	K(0.986)AIEEK(0.014)EEGLDTFVVQDGTNWSTGQR	Up
XP_024176720.1	632	ABC transporter C family member 10-like	SASFSWDNLSK(1)ATLR	Up
XP_024189449.1	725	Putative ABC transporter C family member 15 isoform X1	ENILFGNAYDK(1)AK	Up
XP_024165536.1	194	Bidirectional sugar transporter SWEET1-like isoform X2	QQPSAEDNVELGLEK(1)PHQSK	Down
XP_024191501.1	1219	ABC transporter D family member 1 isoform X2	LYQEGGK(1)FDDSTNILDMR	Down
XP_024181861.1	1066	ABC transporter B family member 19	FYDPIVGK(1)VMIDGK	Down
XP_024158774.1	1478	ABC transporter C family member 2-like	SAFSK(1)MVQSTGAANAQYLR	Down
XP_024194123.1	478	Ammonium transporter 1 member 1	HGGFAYVYHDEDDAGK(1)PAGIQLR	Down
XP_024165085.1	332	Lysine histidine transporter-like 8	GHNLILEIQATMPSSEK(1)HPSR	Down
XP_024176551.1	64	Copper transport protein ATX1-like	K(1)TAYWEAEAPAEPEAK	Down
XP_024169378.1	441	Molybdate-anion transporter-like	LFVITDGHK(1)SK	Down
XP_024194192.1	4	Aluminum-activated malate transporter 4	AAK(1)IGSFR	Down
XP_024163410.1	64	ABC transporter E family member 2	K(1)CPFEAIQIINLPK	Down
XP_024165658.1	153	Zinc transporter 4, chloroplastic-like	K(1)QGLARPTEDQVR	Down
XP_024166097.1	99	Amino acid transporter AVT3B-like	KLESPDAPTK(1)IASFGDLGFR	Down
XP_024176551.1	46	Copper transport protein ATX1-like	VTVK(1)GNVPPETVLQTVTK	Down
XP_024176711.1	886	ABC transporter C family member 10-like	GDQLIK(1)LEER	Down
XP_024180604.1	145	Potassium transporter 4	YGPSSQVAASSPLK(1)R	Down
XP_024180746.1	363	ABC transporter G family member 11-like	SYK(1)SSENYHQLQR	Down
XP_024181557.1	442	Copper transport protein CCH-like	DTIPSNFQK(1)R	Down
XP_024186868.1	1208	ABC transporter B family member 1	FVSALPDGYK(1)TFVGER	Down
XP_024186868.1	731	ABC transporter B family member 1	LEK(1)LAFK	Down
XP_024186868.1	66	ABC transporter B family member 1	K(0.824)ESNDSGGGEK(0.176)PEAVPSIGFGEVFR	Down
XP_024186868.1	76	ABC transporter B family member 1	ESNDSGGGEK(1)PEAVPSIGFGEVFR	Down
XP_024192899.1	4	Sucrose transport protein SUC3	AGK(1)TDSVSIR	Down
XP_024193193.1	198	Putative potassium transporter 12	LKLPTPELK(1)R	Down
XP_024194123.1	486	Ammonium transporter 1 member 1	K(1)VEPNSSTPNSV	Down
XP_024198608.1	337	ABC transporter F family member 1	FGHGSAK(1)LAR	Down
XP_024198608.1	192	ABC transporter F family member 1	LEALDAATAEK(1)R	Down
XP_024198608.1	325	ABC transporter F family member 1	WEQEQIANMK(1)EYIAR	Down
XP_024177255.1	253	Probable aquaporin PIP-type 7a	SLGAAIIFNK(1)DR	Down
XP_024177255.1	14	Probable aquaporin PIP-type 7a	MQAK(1)EEDVSLGANK(1)FPER	Down
XP_024175740.1	14	Aquaporin PIP1-3	LGANK(1)FSER	Down
XP_024166688.1	269	Aquaporin PIP2-7	AAAIK(1)ALGSFR	Down
XP_024166688.1	3	Aquaporin PIP2-7	TK(1)EVSEEPQAHHHK	Down
XP_024193331.1	204	Auxin efflux carrier component 3-like PIN3-like	DFLETDAEIGDDGK(1)LHVK	Down

## Supplementary Materials

Supplementary materials can be found at https://www.mdpi.com/1422-0067/20/24/6108/s1.
